# Gut microbiota interactions with the immunomodulatory role of 25-hydroxyvitamin D in children with infectious mononucleosis

**DOI:** 10.3389/fmed.2026.1755236

**Published:** 2026-01-28

**Authors:** Xiangyu Chen, Shanshan Huang, Jiandong Shi

**Affiliations:** 1 The Ward I of Pediatric, Jinhua Maternal and Child Health Care Hospital, Zhejiang, China; 2 The Affiliated Jinhua Hospital of Wenzhou Medical University, Zhejiang, China; 3 Department of Hematology, Yuyao People’s Hospital of Zhejiang Province, Ningbo, Zhejiang, China

**Keywords:** 25-hydroxyvitamin D, cytokine, Gutmicrobiota, infectiousmononucleosis, lymphocyte subsets

## Abstract

**Introduction:**

Infectious mononucleosis (IM) is a childhood infectious disease caused by the Epstein–Barr virus (EBV). Its pathogenesis is associated with immune and gut microbiota disorders. Exploring the regulatory factors of immune function and gut microbiota in IM may provide novel strategies for the prophylaxis and management of this condition. This study explored the association between 25-hydroxyvitamin D (25(OH)D) level and gut microbiota abundance in children with IM.

**Methods:**

Individuals meeting the inclusion criteria for IM were enrolled, and their blood samples were collected. The clinical manifestations, 25(OH)D levels, cytokines, and lymphocyte subsets were selected for correlation analysis. A total of 99 participants were divided into three groups, based on the level of 25(OH)D. Sixty participants completed fecal collection, including those with suboptimal 25(OH)D (*n* = 29) and optimal 25(OH)D (*n* = 31). The V3–V4 region of the 16S rRNA gene of fecal microbiota was sequenced, bioinformatics analysis was performed, and the association between the relative abundances and 25(OH)D level was analyzed.

**Results:**

The 25(OH)D level was positively correlated with the percentages of NK cells and B cells, and negatively correlated with IL-1*β*, IL-12, IL-17, TNF-*α*, T cells, and CTL cells (all *p* < 0.01). Compared with the optimal 25(OH)D group, the 25(OH)D suboptimal group showed higher relative abundances of *Bacillota* and *Proteobacteria,* and lower relative abundances of *Bacteroidetes* and *Actinobacteria*r. Compared with the optimal group, the relative abundances of *Acinetobacter* (LDA = 2.53, *p* = 0.02), *Epulopiscium* (LDA = 2.76, *p* = 0.02), and *Collinsella* (LDA = 3.16, *p* = 0.04) increased, while the relative abundances of *Anaerostipes* (LDA = 2.76, *p* = 0.042), *Helicobacter* (LDA = 2.79, *p* = 0.041), and *Desulfovibrio* (LDA = 2.76, *p* = 0.02) decreased in suboptimal group. *Anaerostipes* was positively correlated with 25(OH)D level (*r* = 0.335, *p* < 0.001), whereas *Acinetobacter* was negatively correlated (*r* = −0.303, *p* < 0.001).

**Conclusion:**

The level of 25(OH)D was associated with gut microbiota and immune function in children with IM. Improving vitamin D deficiency may help maintain normal gut microbiota, ameliorate immune dysregulation, and reduce IM-related complications.

## 1 Introduction

Infectious mononucleosis (IM) is an infectious disease in children caused by Epstein–Barr virus (EBV), which can manifest as isthmitis, fever, hepatosplenomegaly, and other syndromes (1). If immune function is abnormal, IM can progress to liver dysfunction, hemophagocytic syndrome, and other complications. At present, IM complications have been found to be associated with patient cytokine levels and the proportion of lymphocyte subsets (2, 3), and they also cause alterations in gut microbiota (4). Because antiviral drugs that inhibit EBV replication and infection are still lacking, it is critical to prevent the development of IM and its associated complications. Gut microbiota preparations can modulate intestinal immune function by altering the abundance of specific microbial taxa, which in turn ameliorates systemic immune dysregulation. Consequently, targeting the crosstalk between immune function and gut microbiota in children with IM may represent a promising novel strategy for prophylaxis and clinical management of this disease.

Vitamin D is an essential nutrient for children’s growth and development and also plays an important role in immunomodulation and infection resistance (5). 1,25-dihydroxy vitamin D (25(OH)D) is stable and has a long half-life, serving as the primary active form of vitamin D in the bloodstream (6). Extensive research confirms serum 25(OH)D exhibits complex immunomodulatory activities. Furthermore, 25(OH)D induces autophagy in monocytes and mediates innate immunity. It also regulates Toll-like receptors to influence proinflammatory cytokine release, thereby increasing complications in infectious diseases (7, 8). 25(OH)D levels are associated with the onset and severity of immune-mediated diseases in inflammatory and damaging conditions (9–13). Current research indicates that serum 25(OH)D levels in pediatric inflammatory bowel disease patients are significantly lower than in healthy individuals, with potential associations to disease duration and organ damage severity (14, 15). As an essential routine nutrient, 25(OH)D has recognized positive effects in regulating immune function, enhancing protection against infectious diseases, and improving autoimmune conditions.

The molecular mechanisms underlying vitamin D’s regulation of gut microbiota and immune response involve multiple pathways. On the one hand, vitamin D binds to its nuclear receptor (VDR) to regulate the expression of target genes, thereby modulating the composition and function of gut microbiota (16). Vitamin D can promote the proliferation of beneficial bacteria that produce short-chain fatty acids (SCFAs), while inhibiting the overgrowth of pathogenic bacteria (17, 18). Meanwhile, 25(OH)D upregulates the expression of intestinal tight junction proteins to enhance the integrity of the intestinal barrier (19), reducing the translocation of intestinal bacteria and their metabolites, which in turn alleviates systemic inflammation. On the other hand, regarding the immune response to infection, 25(OH)D modulates the functions of both innate and adaptive immune cells and enhances the phagocytic capacity of macrophages and the antigen-presenting function of dendritic cells to eliminate pathogen-infected cells (20, 21). Simultaneously, 25(OH)D suppresses the excessive activation of pro-inflammatory cytokines and reduces the infiltration of inflammatory cells (22).

Our previous research has shown that children with IM exhibit characteristic changes in their gut microbiota, and these changes are associated with immune disorders (23). In this study, we investigated whether characteristic changes in gut microbiota occur in vitamin D insufficiency and how these changes are related to 25(OH)D levels, thus laying the foundation for exploring the role of 25(OH)D in the occurrence and development of IM.

## 2 Patients and study design

### 2.1 Participant recruitment

This was a case–control study that included 99 children with IM between January and December 2024 at Jinhua Maternal and Child Health Care Hospital. Among all 99 IM patients, 29 children were randomly selected from the deficiency and insufficiency groups to form the suboptimal 25(OH)D group. Together with the optimal group, a total of 60 fecal samples were collected for microbiota analysis. This study was approved by the Medical Ethics Committee of Jinhua Maternal and Child Health Hospital (2021KY033). Informed consent was obtained from each patient’s parents, legal guardians, or the patients themselves. The sample size was determined using G*Power 3.1 software.

The diagnosis criteria of IM patients were as follows: (1) Clinical symptoms were consistent with IM manifestations, such as fever, isthmitis, lymphadenopathy, and hepatosplenomegaly; (2) EBV-VCA IgM or EA IgG was positive and anti-EBNA-IgG was negative, EBV-DNA >1×10^(2.5)/mL and/or the serum anti-EBV-VCA-IgG antibody titer increased more than four times; and (3) Peripheral blood atypical lymphocyte ratio ≥0.10 and/or lymphocytosis ≥5.0 × 10^9^/L. The exclusion criteria were as follows: (1) Diarrhea caused by foodborne infection, irritable bowel syndrome, autumn diarrhea, allergic diarrhea, and other causes; (2) Use of antibiotics, probiotics, prebiotics, metformin, glucocorticoids, gut microecological modulators, or immunosuppressants within the past 2 weeks; and (3) Chronic active EBV infection.

### 2.2 Sample collection

All patients eligible for inclusion were required to provide demographic information and previous health status, and to record the eating habits, physical activity (International Physical Activity Questionnaire), and sun exposure during the preceding month. Peripheral blood samples were collected before initial treatment, and all samples were tested within 6 h of collection. Fecal samples were collected within 24 h after IM diagnosis. Fresh fecal samples were collected in sterile stool retention boxes and stored at −80 °C within 30 min.

### 2.3 Physiological index detection

All blood samples were tested in the laboratory of Jinhua Maternal and Child Health Care Hospital on the day of collection. The liver functions, such as alanine aminotransferase (ALT), aspartate aminotransferase (AST), albumin (ALB), total bilirubin (TBil), and renal function (creatinine), were examined using an automatic biochemical analyzer (Hitachi, 7600-020, Tokyo, Japan). Routine blood parameters, including white blood cells (WBC), red blood cells (RBC), platelets (PLT), and hemoglobin (Hb), were analyzed using an automated hematology analyzer (Sysmex, XN-10, Kobe City, Japan). The prothrombin time was examined using an automatic blood coagulation analyzer (Werfen, ACLTOP750CTS, Barcelona, Spain). The serum markers of EBV (VCA-IgG, VCA-IgM, VCA-IgA, EA-IgA, and EBNA-IgG) were analyzed using an automatic chemiluminescence analyzer (Biocup, 20,173,404,075, Shenzhen, China). The EBV-DNA load was examined using a gene amplification instrument and its supporting reagent (Daan Gene, 20,233,400,997, Guangzhou, China). Specific enzyme-linked immunosorbent assay techniques were used, according to the manufacturer’s instructions (Biosource, Camarillo, California, USA), to determine the serum levels of the following cytokines: interleukin-1β, (IL-1β), interleukin-2, (IL-2), interleukin-4, (IL-4), interleukin-5 (IL-5), interleukin-6 (IL-6), interleukin-8 (IL-8), interleukin-10 (IL-10), interleukin-12 (IL-12), interleukin-17 (IL-17), tumor necrosis factor-alpha (TNF-*α*), interferon-gamma (IFN-*γ*), and interferon-alpha (IFN-α). Cytokine concentrations were determined spectrophotometrically, and the absorbance was read at 450 nm (BioTek, Vermont, USA). Lymphocyte subsets, including T cells, natural killer cells, helper T cells, cytotoxic T cells, double-negative T cells, and B cells, were detected using flow cytometry. Briefly, the absolute counts (cells/μL) of CD3^+^T cells, CD3^+^CD4^+^T cells, CD3^+^CD8^+^T cells, CD19^+^B cells, and CD3^−^CD16^+^CD56^+^natural killer cells were quantified using multi-color flow cytometry. Staining was performed with a panel of human monoclonal antibodies: anti-CD3-fluorescein isothiocyanate (FITC), anti-CD4-phycoerythrin (PE), anti-CD8-allophycocyanin (APC), anti-CD19-PE, anti-CD16-APC, and anti-CD56-PE (BD Multitest, New Jersey, USA), following the manufacturer’s protocols. Data acquisition and analysis were conducted on a BD FACS Canto II flow cytometer (BD Biosciences, New Jersey, USA). The serum 25(OH)D level was detected by liquid chromatography–tandem mass spectrometry. According to the serum level of 25(OH)D, the patients were divided into optimal 25(OH)D group [25(OH)D ≥ 30 μg/L] and suboptimal 25(OH)D group [25(OH)D < 30 μg/L], including insufficiency group [20 μg/L ≤ 25(OH)D < 30 μg/L] and deficiency group [25(OH)D < 20 μg/L] (24). All samples were centrifuged and kept in the dark at room temperature for 15 min. Each sample was analyzed using a multi-color flow cytometer (BD FACSCanto II, New Jersey, USA) according to the manufacturer’s instructions.

### 2.4 DNA extraction and 16S rRNA sequencing analysis

Total genomic DNA was extracted from the fecal samples using the cetyltrimethylammonium bromide method. According to the selection of the sequencing region (the 16S V3–V4 region), the barcode-specific primers were used for polymerase chain reaction (PCR) amplification. The PCR products were purified by 2% agarose gel electrophoresis, and sequences with the main strip of 400–450 bp were chosen for further experiments. The sequencing libraries were generated using an Illumina TruSeq DNA PCR-Free Library Preparation Kit (Illumina, USA). The library quality was assessed with a Qubit @ 2.0 Fluorometer (Thermo Scientific) and an Agilent Bioanalyzer 2,100 system. Finally, the libraries were sequenced on an Illumina NovaSeq platform. The *α*-diversity indices (Chao1 and Simpson) were calculated using QIIME software (version 1.9.1). The *β*-diversity was evaluated using unweighted UniFrac metrics and principal coordinate analysis to assess the microbial structure and distribution. The Venn diagram was drawn using R software (version 4.3.1). The linear discriminant analysis (LDA) effect size (LEfSe) was applied to identify different species between groups, with a log LDA score > 2 set as the threshold of differential taxa.

### 2.5 Statistical analysis

All statistical analyses were performed using R software (version 4.3.1). Descriptive data are expressed as mean and standard deviation. Comparisons among the deficiency, insufficiency, and optimal 25(OH)D groups were conducted using a one-way analysis of variance (ANOVA) to assess differences in the dependent variables across the three independent groups, examining whether the group means differed significantly by comparing the between-group variance to the within-group variance. Following a significant one-way ANOVA, *post-hoc* Bonferroni correction was applied to conduct pairwise comparisons between groups. The *F*-value was used to determine the significance of differences between groups, and an adjusted *p* < 0.0001 was considered statistically significant. The associations between 25(OH)D and immunomodulatory role were analyzed using the Pearson’s correlation coefficient (two-tailed) test. A *p*-value < 0.05 indicated statistical significance.

## 3 Results

### 3.1 Subject characteristics

A total of 99 samples were included, including 57 male (57.6%) and 42 female patients (42.4%). The mean age was 5.62 ± 2.71 years. All patients were divided into three groups: deficiency (*n* = 25), insufficiency (*n* = 43), and optimal (*n* = 31) 25(OH)D groups. Significant differences were observed among the three groups in IL-12 (*F* = 9.786, *p* < 0.0001), T cell % (*F* = 11.142, *p* < 0.0001), and B cell % (*F* = 8.419, *p* < 0.0001). In the optimal 25(OH)D group, the percentage of T cells was lower than that in the 25(OH)D insufficiency group, whereas the percentage of B cells was higher than in both the 25(OH)D insufficiency and 25(OH)D deficiency groups. Additionally, the level of IL-12 in the 25(OH)D insufficiency group was lower than the 25(OH)D deficiency group. The clinical characteristics of patients are shown in Table 1.

**Table 1:** General demographic data and clinical characteristics.

Indicators	25(OH)D deficiency (*n* = 25)	25(OH)D insufficiency (*n* = 43)	Optimal 25(OH)D (*n* = 31)	*F*	*p*
Sex (F/M)	12/13	20/23	10/21	–	–
Age (year)	5.57 ± 1.64	5.38 ± 2.19	5.93 ± 2.21	0.55	0.587
Fever time (days)	4.63 ± 3.40	3.48 ± 2.34	4.11 ± 2.74	1.311	0.275
WBC (×10^9^/L)	13.80 ± 4.93	12.92 ± 3.47	13.93 ± 5.36	0.53	0.59
Lym (×10^9^/L)	16.38 ± 20.37	8.90 ± 3.04	9.44 ± 5.44	4.023	0.021
Abnormal Lym%	11.21 ± 5.21	12.43 ± 5.72	12.88 ± 5.65	0.518	0.597
ALT (U/L)	81.05 ± 75.77	82.82 ± 81.75	76.47 ± 76.72	0.602	0.550
AST (U/L)	62.42 ± 38.89	76.50 ± 63.93	59.22 ± 47.75	0.969	0.384
EB-DNA (×10^5^)	265,161,363 ± 231,016.61	1,081,231.63 ± 5,515,329.09	533,601.78 ± 798,229.46	0.346	0.708
Ferritin	86.47 ± 56.57	76.28 ± 32.26	71.63 ± 24.05	0.928	0.399
IL-1*β* (pg/mL)	3.66 ± 2.24	2.96 ± 2.61	2.05 ± 1.53	2.975	0.056
IL-2 (pg/mL)	3.20 ± 1.99	2.19 ± 1.49	2.58 ± 2.42	1.859	0.162
IL-4 (pg/mL)	3.54 ± 2.10	2.57 ± 1.70	3.44 ± 2.18	2.453	0.092
IL-5 (pg/mL)	1.20 ± 0.67	0.90 ± 0.58	1.16 ± 0.71	2.151	0.123
IL-6 (pg/mL)	20.96 ± 13.76	18.02 ± 11.07	14.31 ± 8.10	2.968	0.057
IL-8 (pg/mL)	35.80 ± 20.06	26.68 ± 12.37	49.79 ± 37.88	3.662	0.03
IL-10 (pg/mL)	44.97 ± 42.09	31.32 ± 17.47	40.03 ± 36.93	1.553	0.217
IL-12 (pg/mL)	5.39 ± 2.98	3.98 ± 2.72^a^	3.89 ± 2.70	9.786	<0.0001
IL-17 (pg/mL)	31.52 ± 18.68	18.68 ± 14.75	18.36 ± 19.91	4.445	0.018
TNF-*α* (pg/mL)	3.49 ± 1.78	2.11 ± 2.00	2.04 ± 1.74	4.192	0.018
*γ*-IFN (pg/mL)	11.68 ± 13.15	7.21 ± 3.96	8.84 ± 6.31	2.402	0.097
*α*-IFN (pg/mL)	5.07 ± 3.50	4.36 ± 6.76	3.92 ± 3.60	0.255	0.775
T (%)	87.64 ± 5.71	85.47 ± 4.92	81.77 ± 7.17^a^	11.142	<0.0001
NK (%)	5.64 ± 3.15	7.02 ± 3.61	8.60 ± 4.40	3.520	0.034
Th (%)	16.25 ± 11.14	15.69 ± 8.63	17.63 ± 9.16	0.363	0.696
CTL (%)	68.11 ± 9.32	62.53 ± 11.57	59.14 ± 1.24	3.828	0.026
CD4+/CD8+	0.25 ± 0.17	0.29 ± 0.24	0.32 ± 0.18	0.652	0.523
DNT (%)	0.31 ± 0.52	0.13 ± 0.96	0.15 ± 0.16	3.677	0.029
B (%)	5.53 ± 2.96	5.76 ± 3.01	8.31 ± 4.34^a, b^	8.419	<0.0001

WBC, white blood cell; Lym, lymphocyte; ALT, alanine aminotransferase; IL, interleukin; TNF, tumor necrosis factor; IFN, interferon; T, T cells; NK, natural killer cells; Th, helper T cells; CTL, cytotoxic T cells; DNT, double-negative T cells; CD4CD8, the ratio of CD4/CD8; B, B cells; IM, infectious mononucleosis; *F*, *F*-value; ^a^Compared with 25(OH)D deficiency, *p* < 0.0001; ^b^Compared with 25(OH)D insufficiency, *p* < 0.0001. Data are presented as mean ± standard deviation (mean ± SD).

### 3.2 Relationship between 25(OH)D and immunomodulatory role

**Figure 1:**
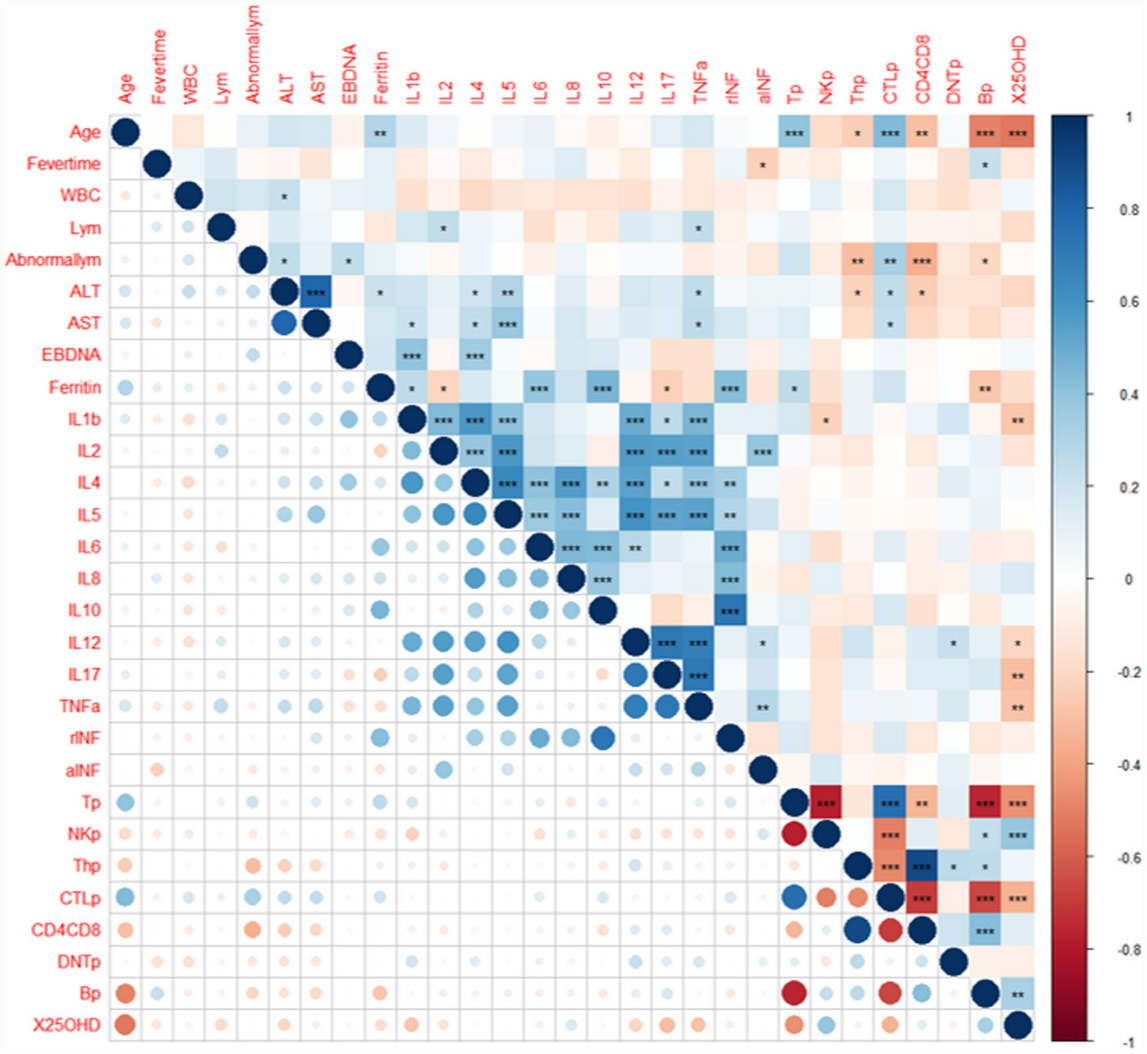
The serum 25(OH)D level was closely related to multiple clinical indicators. The serum 25(OH)D level positively correlated with the NK cells% and B cells% and negatively correlated with IL-1*β*, IL-12, IL-17, TNF-*α*, T cells%, and Ts cells%. WBC, white blood cell; Lym, lymphocyte; ALT, alanine aminotransferase; IL, interleukin; TNF, tumor necrosis factor; IFN, interferon; Tp, T cells %; NKp, natural killer cells %; Thp, helper T cells %; CTLp, cytotoxic T cells %; DNTp, double-negative T cells %; CD4CD8, the ratio of CD4/CD8; and Bp, B cells %. X25OHD, 25-hydroxyvitamin D, ^*^p < 0.05, ^**^p < 0.001, ^***^p < 0.0001.

A correlation analysis was performed between 25(OH)D levels and clinical indicators to better understand the relationship between 25(OH)D and its immunomodulatory role. Serum 25(OH)D levels positively correlated with the percentage of NK cells (*r* = 0.38, *p* = 1.93 × 10^−4^) and B cells (*r* = 0.32, *p* = 2.09 × 10^−3^), and negatively correlated with the IL-1*β* (*r* = −0.27, *p* = 8.78 × 10^−3^), IL-12 (*r* = −0.21, *p* = 4.48 × 10^−2^), IL-17 (*r* = −0.31, *p* = 3.17 × 10^−3^), TNF-*α* (*r* = −0.28, *p* = 6.82 × 10^−3^), T cells (*r* = −0.45, *p* = 8.42 × 10^−6^), and CTL cells (*r* = −0.34, *p* = 9.05 × 10^−4^) (Figure 1).

### 3.3 Differences in microbial community structure between suboptimal and optimal 25(OH)D groups

**Figure 2:**
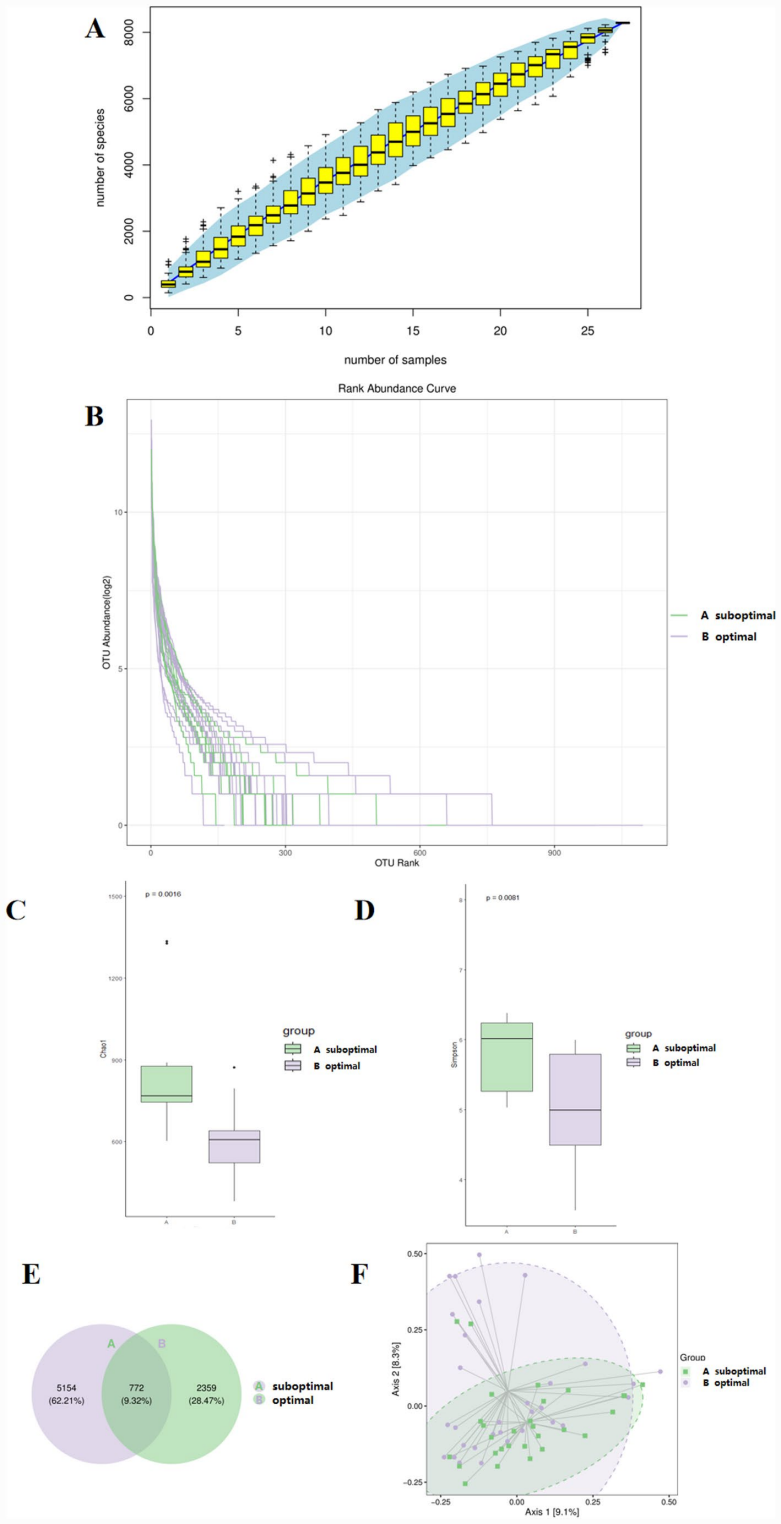
Comparison of gut microbiota diversity between the suboptimal and optimal 25(OH)D. **(A)** The rarefaction curve tends to be flat, indicating that the sequencing depth was sufficient. **(B)** The rank abundance curve tends to be flat, indicating even species distribution. **(C)** The Chao1 index and **(D)** The Simpson index significantly decreased in 25(OH)D suboptimal. **(E)** The Venn diagram showed that 772 OTUs were shared between the suboptimal and optimal 25(OH)D, and 5,154 OTUs were unique to the 25(OH)D suboptimal group. **(F)** Principal coordinate analysis of *β*-diversity of PCoA analysis based on the Jaccard metric showed that the samples with suboptimal 25(OH)D levels were farther away from optimal 25(OH)D levels.

**Figure 3:**
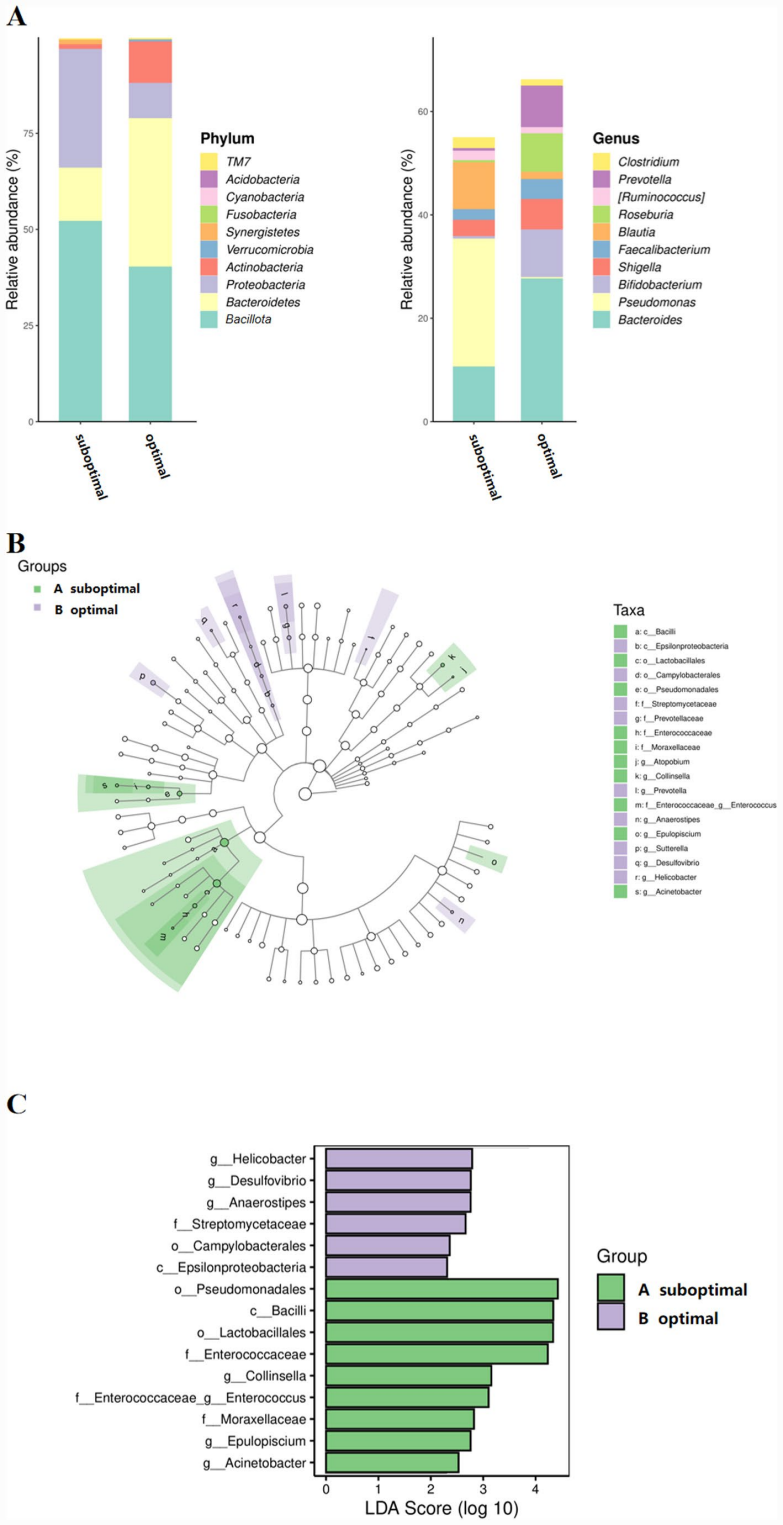
Characteristics of gut microbiota of suboptimal and optimal 25(OH)D. The relative abundances of gut microbiota between the suboptimal and optimal 25(OH)D groups at the phylum **(A)** and genus level **(B)**. **(C)** Differential taxa identified by the LEfSe analysis between the suboptimal and optimal 25(OH)D groups (LDA > 4.0).

There were 29 children with suboptimal 25(OH)D levels and 31 children with optimal 25(OH)D levels. All the samples were clustered into 8,285 OTUs. The rarefaction curve and the rank abundance curve in each group tended to be flat, indicating that the sequencing depth was sufficient and the sample species distribution was uniform (Figures 2A,B). The *α*-diversity of gut microbiota, assessed by Chao1 and Simpson indices, increased in the suboptimal 25(OH)D group compared with the optimal 25(OH)D group (Figures 2C,D). The Venn diagram showed that 772 OTUs were shared between the two groups, and 5,154 OTUs were unique to the suboptimal 25(OH)D group (Figure 2E). The *β*-diversity of gut microbiota was calculated based on Jaccard metrics and principal coordinate analysis, which showed that the samples of gut microbiota in the suboptimal and optimal 25(OH)D groups were clustered (Figure 2F). At the phylum level, the relative abundances of *Bacillota* and *Proteobacteria* in suboptimal 25(OH)D were higher than in the optimal 25(OH)D group, whereas the relative abundances of *Bacteroidetes* and *Actinobacteria* were lower. The relative abundances of *Bacteroidetes* in the suboptimal and optimal 25(OH)D groups was 14.37 and 38.65%, respectively; *Bacillota* in the suboptimal and optimal 25(OH)D groups was 52.17 and 40.45%, respectively; and *Proteobacteria* in the suboptimal and optimal 25(OH)D groups was 30.8 and 9.3%, respectively (Figure 3A). At the genus level, compared with the optimal 25(OH)D groups, the abundances of *Pseudomonas* and *Blautia* in the suboptimal 25(OH)D increased, and the abundances of *Bacteroides* and *Bifidobacterium* in the suboptimal 25(OH)D decreased. The characteristics of gut microbiota between the suboptimal and optimal 25(OH)D groups were analyzed using the LEfSe method. Compared with the optimal 25(OH)D group, the relative abundances of *Acinetobacter* (LDA = 2.53, *p* = 0.02), *Epulopiscium* (LDA = 2.76, *p* = 0.02), and *Collinsella* (LDA = 3.16, *p* = 0.04) increased, while the relative abundances of *Anaerostipes* (LDA = 2.76, *p* = 0.042), *Helicobacter* (LDA = 2.79, *p* = 0.041), and *Desulfovibrio* (LDA = 2.76, *p* = 0.02) decreased in the suboptimal 25(OH)D group (Figures 3B,C).

**Figure 4:**
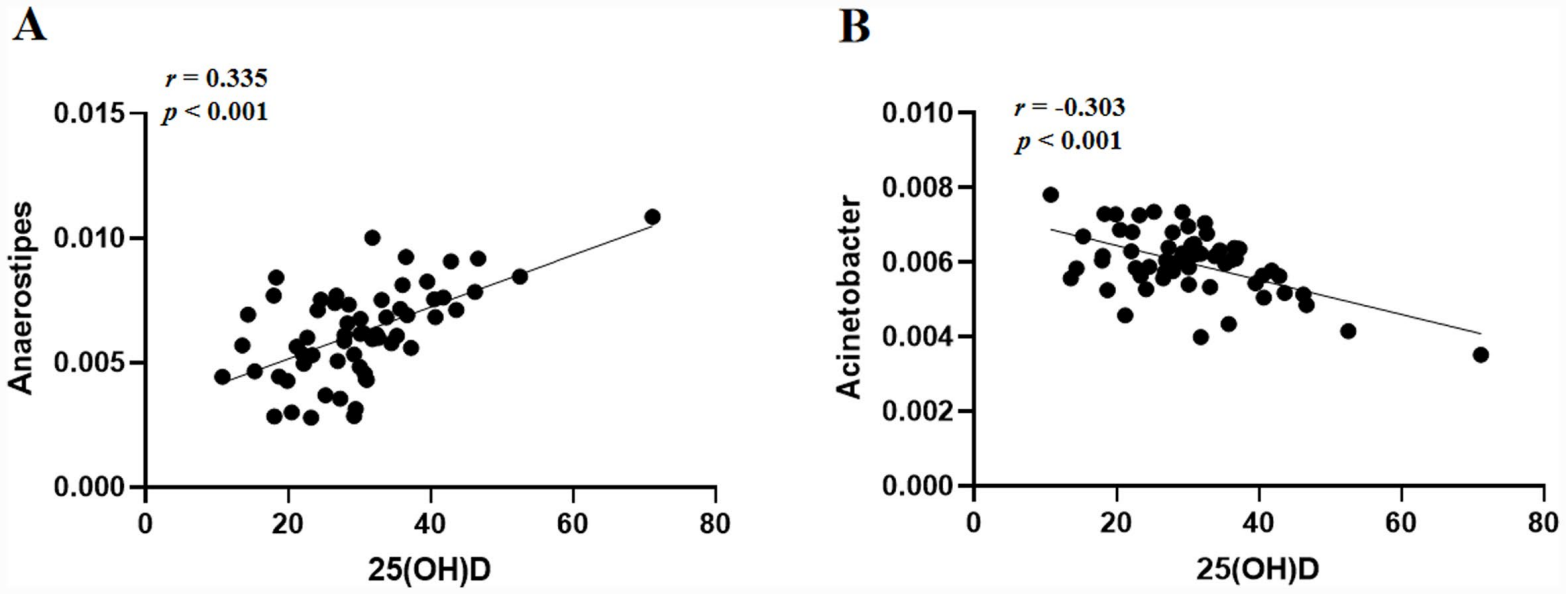
Association between the serum 25(OH)D level (μg/L) in children with IM and the relative abundances of *Anaerostipes* and *Acinetobacter*. **(A)** *Anaerostipes* and **(B)** *Acinetobacter*. *r* denotes Pearson’s correlation coefficient.

Correspondingly, *Anaerostipes* was found to be positively correlated with the 25(OH)D level (Pearson’s correlation, *r* = 0.335, *p* < 0.001, Figure 4A). *Acinetobacter* was found to be negatively correlated with 25(OH)D level (Pearson’s correlation, *r* = −0.303, *p* < 0.001, Figure 4B). However, there was no association with 25(OH)D levels in *Epulopiscium*, *Collinsella*, *Helicobacter*, and *Desulfovibrio* across children with IM (*p* > 0.05).

## 4 Discussion

IM is a common infectious disease caused by EBV infection, with a predominant occurrence in preschool and school-aged children (25). EBV primarily invades and replicates in B lymphocytes due to the presence of EBV-LMP receptors on human B cells (26). EBV infection of B cells activates cytotoxic T cells, leading to an increase in heterotypic lymphocytes and a massive release of inflammatory factors, resulting in histopathologic changes in multiple organs and a series of clinical symptoms (27). Previous studies have focused on the association between immune disorders and IM. Although many observational studies have been performed to explore the link between immune disease and IM, few have investigated the influence on EBV disease progression through immune function modulation. The gut microbiota can affect the inflammatory cells of the intestinal lamina propria and, via the enterohepatic axis, influence the immune system. Our previous studies have found alterations in the diversity and specific bacterial abundance of EBV-infected children. Thus, exploring nutrients that jointly affect immune function and gut microbiota disorders could be a novel strategy in the prophylaxis and management of IM (23).

Vitamin D, a fat-soluble vitamin involved in bone and mineral metabolism, is synthesized primarily in the skin under UVB radiation (28). The main form of vitamin D in the circulatory system is 25(OH)D. 25(OH)D undergoes further hydroxylation by the cytochrome P450 enzyme CYP27B1 to form 1,25-dihydroxyvitamin D [1,25(OH)_2_D], the biologically active metabolite. 1,25(OH)_2_D enters cells through vitamin D receptors and participates in various cellular processes (29). Vitamin D receptors are widely present in immune system cells, suggesting that vitamin D may induce immune tolerance, and deficiency may be associated with autoimmune disease occurrence (9, 30). In addition, reduced 25(OH)D levels are currently observed in autoimmune diseases such as atopic dermatitis, rheumatoid arthritis, multiple sclerosis, and systemic lupus erythematosus (31). A clinical retrospective study showed that 25(OH)D levels were lower in patients with acute IM than in healthy controls and that 25(OH)D levels were negatively correlated with viral titers (14). Vitamin D deficiency may increase viral susceptibility, though the mechanism remains unclear. Munger reported that low levels of 25(OH)D modulated the immune response to EBV infection, and supplementation with vitamin D reduced salivary shedding of EBV (32). T-regulatory (T-reg) cells may play a key role in the relationship between vitamin D and EBV infection. Vitamin D may control cellular function and immune cell proliferation by regulating gene expression, mediating the Th-2 response, inhibiting the Th-1 response, and inducing T-reg cells (33). However, it has also been shown that there is no correlation between VCA IgM and 25(OH)D levels in acute EBV infection, suggesting that vitamin D and EBV may play independent roles in IM (15). Therefore, further exploration is needed to evaluate the interaction between 25(OH)D and EBV, the causal association of vitamin D deficiency with IM development, and whether vitamin D supplementation independently improves IM prognosis.

Our clinical cohort study found that 25(OH)D levels were positively correlated with the percentage of NK cells and B cells and negatively correlated with IL-1*β*, IL-12, IL-17, TNF-*α*, T cells, and CTL cells. Children with low 25(OH)D levels have abnormal lymphocyte ratios, decreased NK cell and B cell ratios, and decreased ability to monitor and eliminate EBV. Higher IL-1*β* and TNF-*α* pro-inflammatory factors may promote EBV-induced immune damage and increase the risk of complications. Notably, the molecular mechanisms underlying the interaction between vitamin D and inflammatory cytokines are further supported by in silico and clinical evidence. A recent study by Massi et al. (34) demonstrated that 25(OH)D forms stable complexes with IL-6 and IL-10 through molecular docking and molecular dynamics simulations, indicating direct molecular interactions between vitamin D and key inflammatory cytokines that modulate the immune response. Although our study focused on IL-1β, IL-12, and TNF-α, the conserved regulatory role of vitamin D in suppressing pro-inflammatory cytokine production is consistent with their findings—specifically, the active metabolite of vitamin D inhibits the expression of type 1 pro-inflammatory cytokines, including IL-6, TNF-*α*, and IL-12, thereby attenuating excessive Th1 immune responses (34, 35). Moreover, Massi et al. (34) observed that in quartile-based analyses, higher vitamin D levels were associated with reduced IL-6 and stable IL-10 levels, reinforcing that vitamin D exerts a dose-dependent regulatory effect on the balance between pro-inflammatory and anti-inflammatory cytokines. Based on previous studies, IL-1*β* levels were found to correlate with the abundance of intestinal *Enterococcus*, *Atopobium,* and *Acinetobacter* flora (9). Therefore, we hypothesized that 25(OH)D levels could influence gut-specific microbiota abundance through cytokine secretion.

Our study results revealed a unique pattern of gut microbiota diversity in children with IM: compared with the optimal 25(OH)D group, the suboptimal group exhibited a significant increase in *α*-diversity. The Venn diagram further demonstrated that the suboptimal group harbored 5,154 unique OTUs, far exceeding the shared OTUs between the two groups, indicating a substantial expansion of microbial taxa in the 25(OH)D-insufficient state. In contrast, β-diversity analysis based on the Jaccard metrics and principal coordinate analysis showed clear clustering of samples within each group, suggesting that 25(OH)D status still drives distinct microbial community structures. This increase in *α*-diversity appears contradictory to conventional views. However, the elevated α-diversity may represent a transient intermediate state of the gut microbiota rather than a stable, balanced community. In chronic inflammatory or metabolic diseases, gut dysbiosis often progresses from an initial “disordered expansion” (elevated α-diversity) to a “diversity collapse” (36). The 5,154 unique OTUs likely include a large proportion of low-abundance, transient taxa that cannot persist long-term; as 25(OH)D insufficiency persists or IM progresses, these taxa may be outcompeted by more virulent pathogens, leading to a subsequent decline in α-diversity. In the context of complex diseases, α-diversity alone is not a sufficient marker of gut health; instead, the combination of taxonomic composition, functional capacity, and clinical correlates is critical to defining dysbiosis.

Recent studies have demonstrated a bidirectional regulatory relationship between 25(OH)D and the gut microbiota. It has been found that 25(OH)D deficiency may exacerbate the pathologic process of infectious diseases by affecting the structure and function of the intestinal flora. Because vitamin D plays an important role in intestinal epithelial antimicrobial peptide expression, macrophage activation, and maintenance of tight junctions between intestinal epithelial cells, vitamin D-deficient patients with an increased number of harmful organisms in the gut microbiome have disturbed intestinal health homeostasis and have a higher risk of recurrence after *Clostridioides difficile* infection (37). In our study, we found an increase in the relative abundances of *Acinetobacter*, *Epulopiscium*, and *Collinsella* in the suboptimal 25(OH)D group, and we hypothesized that 25(OH)D insufficiency may affect immune function through mechanisms such as reduced bacterial diversity and bacterial metabolite interactions. The gut microbiota can influence vitamin D metabolism: commensal bacteria produce hydroxylases that convert inactive 25(OH)D into active 1,25(OH)D. Vitamin D receptors were widely expressed in intestinal epithelial cells and involved in the maintenance of intestinal barrier function and flora homeostasis. Vitamin D insufficiency may disrupt the integrity of the intestinal barrier, leading to the overproliferation of conditionally pathogenic bacteria (*Acinetobacter*) (38) and suppressing the abundance of SCFAs-producing bacteria (*Prevotella, Desulfovibrio*) (39), and the gut imbalance may further exacerbate gut inflammation and promote systemic immune abnormalities after EBV infection. In addition, gut flora may influence host 25(OH)D levels through metabolites (butyrate, bile acids), and vitamin D deficiency may reduce secretion of antimicrobial peptides (defensins), weakening local defenses against pathogens (40, 41). Interestingly, we observed a reduction in the abundance of *Helicobacter* in the suboptimal 25(OH)D group, as this genus is widely recognized as a potential pathogen associated with gastrointestinal mucosal inflammation, epithelial damage, and systemic immune dysregulation (42). This observation contradicts conventional expectations. 25(OH)D deficiency is associated with elevated levels of IL-17, a pro-inflammatory cytokine demonstrated to inhibit *Helicobacter* colonization by enhancing mucosal barrier function and promoting secretion of antimicrobial peptides such as defensins (43). In this study, the reduced *Helicobacter* load in the suboptimal 25(OH)D group aligns with this mechanism: 25(OH)D deficiency upregulates IL-17, which directly inhibits *Helicobacter*.

In our previous study, it was found that the abundance of *Enterococcus*, *Atopobium*, and *Acinetobacter* correlated positively with the levels of IL-1*β*, IL-6, TNF-*α,* and CTL cells %, EBV infection significantly activates the host immune system as evidenced by the expansion of CTL cells, abnormal NK cells activity and elevated levels of pro-inflammatory cytokines (IL-6, TNF-*α*), and enrichment of pro-inflammatory genera in the intestinal flora may promote the release of IL-6 and TNF-*α* through the activation of the monocyte–macrophage system, thereby exacerbating the systemic inflammatory response (23). Correlation analysis further revealed that 25(OH)D levels positively correlated with *Anaerostipes* and negatively correlated with *Acinetobacter*. Vitamin D regulates various immune cells and modulates the microbiome by influencing their activity (44), including IgE, Th cells 1 and Th cells 2, proinflammatory and anti-inflammatory cytokines (45, 46), and maintains the gastrointestinal barrier function. 25(OH)D increases the number of SCFAs-releasing bacteria, which in turn increases the secretion of IgA by SCFAs-exposed B cells. IgA plays an important role in the maintenance of the immune response to the intestinal barrier by decreasing the number of macrophages and dendritic cells and by restricting the chemotactic properties of neutrophils (47). IgA regulated with acetate binds to specific microorganisms that are involved in regulating the inflammatory response by increasing the levels of the anti-inflammatory IL-10 and IL-18, as well as decreasing the levels of pro-inflammatory IL-1, TNF-*α*, IL-6, IL-8, and IL-12 (48, 49). In this study, we found that 25(OH)D levels in children with IM were positively correlated with the proportion of B cells and NK cells and the abundance of butyric acid-producing bacteria and negatively correlated with the pro-inflammatory factors IL-1*β*, IL-12, and TNF-*α*. We hypothesized that during immune dysfunction induced by EBV, 25(OH)D deficiency disrupts the balance of the gut microbiota, and the dysregulated microbiota reduces vitamin D activation, forming a vicious cycle that exacerbates immune inflammation. 25(OH)D may suppress the release of pro-inflammatory factors by regulating lymphocyte proportions. Concurrently, it could enhance the abundance of intestinal butyrate-producing bacteria and inhibit the proliferation of opportunistic pathogens, thereby mitigating the body’s inflammatory response.

This study demonstrates significant associations between 25(OH)D levels, immune dysregulation, and gut microbiota dysbiosis in children with IM. However, several key limitations should be acknowledged. First, as a cross-sectional study, it cannot establish causal relationships among these variables, only associations. Second, the sample size was relatively small, limiting stratified analyses by age, sex, or disease severity, which could have revealed more nuanced relationships. Third, due to the complexity of establishing a reliable EBV infection animal model, we were unable to perform interventional studies to directly test causal hypotheses. To address these limitations, future research should prioritize: (1) conducting large-scale, multicenter longitudinal studies to verify causality and explore temporal relationships; (2) performing stratified analyses to differentiate between patient groups with distinct IM-related complications; (3) utilizing more established EBV-infected animal models and *in vitro* cell models to deeply dissect the interactions between vitamin D, gut microbiota, and EBV-induced immune responses; and (4) randomized controlled trials investigating the efficacy of vitamin D supplementation, alone or in combination with probiotics, on IM outcomes to translate these findings into clinical practice.

## 5 Conclusion

This study confirmed that 25(OH)D levels are associated with lymphocyte disturbances, cytokine levels, and the abundance of intestinal butyrate-producing and pathogenic bacteria in IM. Clinically, routine 25(OH)D screening is recommended for children with IM, with targeted supplementation in cases of deficiency. Future research should verify causality, optimize supplementation regimens, and explore vitamin D–probiotic synergy and the underlying mechanisms.

## Data Availability

The original contributions presented in the study are included in the article/supplementary material, further inquiries can be directed to the corresponding authors.
